# ‘*They hear “Africa” and they think that there can’t be any good services*’ – perceived context in cross-national learning: a qualitative study of the barriers to Reverse Innovation

**DOI:** 10.1186/s12992-015-0130-z

**Published:** 2015-11-19

**Authors:** Matthew Harris, Emily Weisberger, Diana Silver, James Macinko

**Affiliations:** Institute of Global Health Innovation, Department of Surgery and Cancer, Division of Surgery, Imperial College London, 10th Floor, QEQM Building, St Mary’s Hospital, Praed Street, London, W2 1NY UK; Commonwealth Fund, 1 East 75th Street, New York, 10021 USA; Department of Nutrition, Food Studies and Public Health, New York University, 411 Lafayette Street, New York, 10003 USA; UCLA Fielding School of Public Health, 650 Charles E. Young Dr. South, Room 31-235B, Center for Health Sciences, Los Angeles, CA 90095-1772 USA

**Keywords:** Peer review, Bias, Diffusion of innovation, Evidence based medicine

## Abstract

**Background:**

Country-of-origin of a product can negatively influence its rating, particularly if the product is from a low-income country. It follows that how non-traditional sources of innovation, such as low-income countries, are perceived is likely to be an important part of a diffusion process, particularly given the strong social and cognitive boundaries associated with the healthcare professions.

**Methods:**

Between September and December 2014, we conducted eleven in-depth face-to-face or telephone interviews with key informants from innovation, health and social policy circles, experts in international comparative policy research and leaders in Reverse Innovation in the United States. Interviews were open-ended with guiding probes into the barriers and enablers to Reverse Innovation in the US context, specifically also to understand whether, in their experience translating or attempting to translate innovations from low-income contexts into the US, the source of the innovation matters in the adopter context. Interviews were recorded, transcribed and analyzed thematically using the process of constant comparison.

**Results:**

Our findings show that innovations from low-income countries tend to be discounted early on because of prior assumptions about the potential for these contexts to offer solutions to healthcare problems in the US. Judgments are made about the similarity of low-income contexts with the US, even though this is based oftentimes on flimsy perceptions only. Mixing levels of analysis, local and national, leads to country-level stereotyping and missed opportunities to learn from low-income countries.

**Conclusions:**

Our research highlights that prior expectations, invoked by the Low-income country cue, are interfering with a transparent and objective learning process. There may be merit in adopting some techniques from the cognitive psychology and marketing literatures to understand better the relative importance of source in healthcare research and innovation diffusion. Counter-stereotyping techniques and decision-making tools may be useful to help decision-makers evaluate the generalizability of research findings objectively and transparently. We suggest that those interested in Reverse Innovation should reflect carefully on the value of disclosing the source of the innovation that is being proposed, if doing so is likely to invoke negative stereotypes.

## Background

Diffusion of innovation in healthcare is not linear; its pathway is chaotic and thorny [1]. In the most comprehensive review of innovation diffusion in services organizations to date, Greenhalgh et al. [2] compile what is referred to as the “standard” attributes of an innovation ripe for adoption. The ideal innovation has relative advantage, is compatible with the norms of the adopter context, is perceived as uncomplicated with observable benefits and finally, holds potential for reinvention. They also note that the adopter context needs to be ready to change and be receptive to the innovation. Innovations should diffuse more readily if the adopter is large, mature, specialized, and has the resources (human, financial, organizational etc.) for change [2]. Adopters must be willing and ready to accept a new idea.

The diffusion of innovation literature is curiously silent on whether one’s view of the source of an innovation matters in the diffusion process. Greenhalgh et al. [2] mention that the innovator-context should be a ‘legitimate’ source but then does not explain what constitutes ‘legitimate’. The marketing literature has been far more active in examining buyers’ perceptions of product country of origin. Buyers’ perceptions of particular extrinsic informational cues are one of the most widely studied phenomena in the international business, marketing, and consumer behavior literatures [3]. In the marketing industry, there has been extensive exploration of the relationship between consumers’ perception of product country of origin and consumer demand [4]. Much of this literature has focused on the likelihood that a source country will evoke either positive or negative consumer connotations [5–8]. Marketing research shows that when consumers see “Made in [Country],” this affects their evaluation of the product [4]. This Country of Origin (COO) effect has significant influences on perceptions of perceived quality or risk [3, 4]. There is a tendency for consumers to evaluate their own country’s products more favorably, as well as a positive relationship between product evaluations and degree of economic development of the product’s country origin [4]. Bilkey and Nes [4] note that a lower GDP cue is related to lower perceptions of quality and value. Products developed abroad in general are perceived as “riskier” than products developed in one’s own country. US-made products are perceived to be of higher quality than products made in low-income countries (LICs) and that specific brands might be evaluated higher or lower when LIC country of origin was revealed. Overall, country of origin has significant effects on consumer brand attitudes [9] and the country of origin of a product serves as a conflated, stereotyped measure for other product attributes [3]. This parallels behavioral research on stereotypes, which suggests that individuals use category membership as a heuristic basis for judgments without considering more detailed information about the object’s characteristics [10]. Psychologists call stereotypes ‘cognitive structures schema’, and believe that this schema simplifies a complex evaluation of something or someone by quickly processing incoming stimuli based on the presence of a few relevant characteristics [11]. This may be compounded by social and cognitive boundaries between different professions can lead to the “non-spread” of innovations. Communities of practice develop ‘ways of working’ (clinical, intellectual, professional) that can be relatively inaccessible to non-members of the group. McGivern and Dopson, refer to this as ‘epistemic communities’ and is a barrier to the transfer of knowledge, expertise and experience between groups [12].

The Reverse Innovation ‘movement’ explores the barriers to adopting low-income country (LIC) innovations in high-income country (HIC) contexts. It is motivated in part by the rapidly changing global health landscape and has gained traction in the US and UK because the unsustainable growth in healthcare expenditure means that there is likely to be a genuine need to learn from LICs [13]. LICs have developed novel innovations and there are multiple opportunities to learn from LICs, for example around improved surgical procedures [14], approaches to improve long-term outcomes in mental illness [15–19] and improved skill mix with scaled use of community health workers [20–22]. However, there are strikingly few examples where LIC innovations have been explicitly adopted in HICs [23]. Drawing on Roger’s diffusion of innovation theory, DePasse advances a diffusion pathway specifically for Reverse Innovation and emphasize that Reverse Innovation is a particular type of innovation diffusion because the learning is non-traditional, going from a low- to high-income country [24]. They suggest that a type of “crossover” is required, where ideas begin to transition from the LIC to the HIC, but shed little light on how this crossover is carried out other than to say that network structures within the adopter context must be receptive to the innovation [24]. In the context of Reverse Innovation, exploration into individual perceptions of sources that may be considered to be non-traditional would seem to be important, particularly given the strong social and cognitive boundaries associated with the healthcare professions, and yet there is little research that explores actors’ views of the innovation’s country of origin and whether it interferes positively or negatively with the adoption process.

We undertook an exploration of the barriers and challenges to Reverse Innovation in the US between September 2014-June 2015. Here we describe our interviews with nearly a dozen US experts in healthcare innovation, international health policy and health systems, including also experts from other spheres such as education and social policy, to examine whether, in their experience translating or attempting to translate innovations from low-income contexts into the US, the source of an innovation matters to actors in the adopter context.

## Methods

### Sampling

Informants were selected purposefully from institutions and organizations known to have an interest in the Reverse Innovation space, and attention was given to ensure that there was representation from academic, non-profit foundation, health system management and innovation think tanks. To ensure an even wider representation, informants were also identified from both executive and managerial cadres and from those with experience or interest in international policy exchange in diverse disciplinary areas such as healthcare, as well as educational policy and social policy reform. Eleven participants were initially identified and all agreed to participate in the interviews which were conducted in-person or by telephone.

### Data collection

As exploratory research, with a focus on views and experiences, we used an open-ended interview style, loosely guided by some specific areas of interest, allowing for free-flowing conversation and examination of divergent themes [25]. These were to explore experiences of ‘Reverse Innovation’ in the US context, to identify the barriers and challenges from the informant’s point of view, and to enquire specifically also into whether perceptions of the innovator context are important in the adopter context. Informants were first contacted by email and once the research was explained and agreement to participate obtained, interviews were arranged for dates, times and locations of their convenience. All the interviews were audio-recorded and verbatim transcripts were obtained from a commercial transcription company under strict confidentiality. Written informed consent was obtained from all informants. Participants had the right to withdraw their consent at any stage without giving a reason although none exercised this right. Potential participants received no inducement to participate. No identifying information was included in the transcripts and the informant list is kept securely apart from any data in a locked filing cabinet. Transcripts were checked and we removed any identifiable information or references immediately upon receipt by the research team and stored them in password-protected files.

All interviews lasted between thirty minutes to an hour. The research protocol was reviewed by the University Committee on Activities Involving Human Subject and deemed exempt from full ethical review (IRB# 14–10294). Interviews were all conducted by the same researcher (MH), during the period Sept-Nov 2014, and stopped once thematic saturation was reached.

### Analytical strategy

Two researchers (MH and EW) reviewed all the transcripts and using the first four transcripts independently indexed thematic categories using the process of constant comparison indexing both vertically, within the same transcript, and horizontally, across the transcripts [26–29]. These early code structures were then reviewed and revised for four further iterations, enabling the codes to be redefined, merged and retired until a final code structure had been obtained which was used to then code the remaining transcripts. Both researchers reviewed all coded transcripts, all of which had been coded independently using the final agreed code structure and any coding disagreements were resolved through consensus. The interviews generated over two hundred pages of verbatim transcript, which was manually coded. Coded data was organized into themes and sub-themes and the researchers identified patterns within the categorized data at higher levels of abstraction, developing explanatory concepts to link the themes where possible.

## Results

### Research from LICs is discounted early on

Our informants have extensive experience translating, or attempting to translate, innovations from low-income countries into the US, including conditional cash transfer programs, community services, hospital accreditation schemes, and educational policies such as teaching HIV education in schools. Others have many years experience at the executive levels of hospital and broader healthcare systems or teach international health policy. Speaking to their experience working with evidence from low-income countries regarding innovations that they believed would work well in the US, we asked whether they experienced any particular reactions or sentiments during the persuasion process with colleagues or other actors in the US. Informants consistently recognized that the source of the innovation or the evidence seems to matter, and that, in particular, evidence from low-income countries is often discounted early on:

‘I’m certain that if I say, Narayana Heart Hospital [in India] has lower infection rates than in the US, they’ll be very skeptical, but if I were to say that the Hernia Hospital in Montreal has better outcomes and lower costs than any operations here, they would probably be more receptive to that.’ (1^st^ Oct 14 Professor of Law)

‘We had a conversation with a funder about a project saying this model has been implemented in 12 different countries, all of them low income, we think it has a lot of relevance for the US and we’d like to bring it to the US. And they were really nervous, really nervous, really nervous. And then we said, oh by the way, its actually also been adapted to the UK. And all of a sudden they were like, oh, the UK, great. It would probably work here then.’ (1^st^ Oct 14 Manager, Innovation Think Tank)

The differentiated reaction noted above suggests that attitudes towards evidence may change based merely on knowledge of where such evidence is from. Many informants expressed a deep dissatisfaction with this, a sense of injustice or unfairness, frustrating efforts to communicate evidence and experience from low-income countries, which in some cases could hold potential benefits for US populations. These attitudes are important because without receptivity, persuasion of the potential benefits of a new innovation or model becomes much more difficult – breaking down barriers of prior expectation and becoming a process of convincing rather than learning. Informants recognized that it is not a level xplaying field, and that in their experience actors tend to downplay the effectiveness or benefits of an innovation from a low-income country often drawing on any number of reasons to refute or undermine the validity and value of models that were from surprising sources:

‘It was a very expected reaction….“*of course they can do it there. Everything’s cheap*”’ (1^st^ Oct 14 Director, Innovation Think Tank)

### The role and complexity of perceived context similarity

Examining more closely the reasons why these reactions occur, informants noticed that it is not just because the innovator context was foreign, but that it was *different*. More specifically, that it was *perceived* to be different. Informants noted that on the basis of perceived context dissimilarity, the likelihood that actors would be more receptive would decrease:

‘If the innovation is coming from a country that is dissimilar to the system in which it is being donated, or being suggested, to then that’s going to raise a certain set of biases in the receiver that shuts them off – the receptiveness to that.’ (10^th^ Oct 14 Vice President, Innovation Think Tank)

‘I think there’s a very kind of understandable, at least initial, reaction that you want to look to places that are maybe similar to you in economy or population, because that may be where things are most transferable.’ (1^st^ Oct 14 Director, Innovation Think Tank)

‘Canadians look more similar [to the US], many more Americans have been, the visuals in the media are more relatable, and so there’s a likeness that makes people more willing to see that it could be applicable.’ (1^st^ Oct 14 Senior Manager, Innovation Think Tank)

At first sight, this could pose little in the way of complexity. By and large, we can conceive of countries as having, broadly, some similar characteristics and intuitively the basis to looking towards countries that are similar to one’s own seems sound. However, with probing, there also seemed to be very little consensus about how contexts are perceived, and the criteria used to describe them. We asked the informants what were the features of context that people bear in mind when they make decisions about similarity and we could find very little commonality in response. For some, the language and cultural cues seemed important, for others the socio-economic level, or the training of the health professionals was important:

‘I think it probably depends on what domain one is thinking of. We probably would presume, rightly or wrongly, that other countries that speak English are more like us than countries that don’t. I think we would think other rich countries are more like us than countries that are poor.’

(2^nd^ Oct 14, Chief Executive, Health System)

I’m probably assuming income levels, education levels, societal rules, laws and regulations, the role of the Church…things of that nature. I think these are natural things that one measures for growth and development in a society….educational system, law enforcement, what kind of opportunities do people have, are they equal opportunities, how does business function with government, is the system democratic or dictatorial?’ (14^th^ Oct 14 Board member, Innovation Think Tank)

‘The other characteristics that people think of to judge if their context is comparable to anothers….that is an interesting question….there’s something around sort of the training and licensing of providers….’ (1^st^ Oct 14, Manager, Innovation Think Tank)

‘It depends what hat I am wearing. Ask me as a health policy person…well, you’ve got Canada with a very different financing system but practice patterns that don’t look all that different. Australia, mixed public/private system, lots of private insurance. Brazil, because of its reliance on private insurance for its middle and upper classes. Canada, because of its language and proximity. Its an interesting question because I have a little bit of knowledge and the differences are quite real…really quite severe, at every level. (15^th^ Oct 14, Professor, Public Health)

Informants recognized that context similarity is based not on data but on perceived characteristics and that determination of which contexts could be construed as similar to the US depended highly on the criteria that one was considering. Nonetheless, each informant had little difficulty stating, with some confidence, which countries were, in their opinion, similar to the US or completely dissimilar to the US:

‘Oh, I would guess China, Russia, to some extent India, Congo, Nigeria, Venezuela, Argentina, probably, Columbia, Jamaica…there are a number of countries [that are too different to the US to learn from]’ (14^th^ Oct 14, Board member, Innovation Think Tank)

‘I guess I’d say Canada, UK, maybe Japan [are the most similar to the US]. Japan because we shaped so much of its society after World War II. We really rebuilt the society, the political system…and the way of doing business.’ (14^th^ Oct 14, Board member, Innovation Think Tank)

‘People in the United States would look to Canada and the UK as the two….you know, they’re English-speaking; I mean we share a common history and, you know, I think that differences between Canada, the US and the UK are much smaller than they are with, really, any other country…..(14^th^ Nov 14, Vice President, Research foundation)

although including the caveat, no less important, that each person would likely have a different view:

‘Does it matter which country it [an innovation] came from for transportability? I think probably the answer is yes. I think some places are more likely to take up the innovation because it comes from the US. Some places may be more likely because it comes from Europe or from China.’ (28^th^ Oct 14, Professor, Applied Psychology)

### Conflating levels of analysis

Another issue is that there is a conflation of levels of analysis. External validity or generalizability of an intervention is a poorly defined construct as it is, and depends on whether the causal mechanisms that explain the interaction between an intervention and an outcome in one context are present in another. Interventions happen locally, requiring change agents, leaders and managers, in local services. This nuance is lost, however, when assumptions concerning the causal mechanisms at a local level are conflated into national level characteristics. Our informants describe a process where actors seem to be drawing on country-level general characteristics to explain causal mechanisms at the local level and then to draw conclusions as to whether these causal mechanisms exist in their own context. The success of an intervention locally is unlikely to be, singularly, because of the GDP per capita of the country, and yet the GDP per capita of the country, or other general mental image, is used to ‘make the leap of faith’ about whether this intervention would work in one’s own context:

‘If you imagine [the] infrastructure, like roads, and schools, and governance systems look like yours, then you can make the leap of faith that the medical systems are similar enough…you could picture a school in Canada being like a school in the US….you sort of believe that its got the same active ingredients that make it feel more similar to the US.’ (1^st^ Oct 14, Senior Manager, Innovation Think Tank)

‘I think part of it is preconceptions about how generalizable things are. There are parts of North Carolina that if you looked at a population, the demographics would look almost entirely the same, or with lower income groups, than parts of Kenya, parts of India. When you generalize [though]…of course the thing to say is that the [innovation] from Canada is going to be more like what we would use and we would need.’ (1^st^ Oct 14, Director, Innovation Think Tank)

This is a mental shortcut that is probably pervasive, and likely to be more pronounced for countries towards which we either hold strong prior attitudes or know very little about. Low-income countries, on the whole, fit this category well because, within the US at least, it is likely that actors will be less familiar with such contexts and rely therefore on media representations that are, if anything, presenting skewed and outdated images:

‘I don’t think that many individuals in this country are well travelled enough to believe that those parts of the world have health systems that could be operating with a degree of functional excellence that could make them relevant….If you have never been to India, you will have an outdated view of it, from National Geographic.’ (1^st^ Oct 14, Senior Manager, Innovation Think Tank)

Succinctly summarized by the following narrative, the informant’s observation that ‘they hear “Africa” and they think there can’t be any good services’ is a worrying exposé of the stereotypes occurring on a frequent basis. Needless to say that not only is Africa not homogeneous, but it also comprises hot spots of innovation that out-perform high-income countries:

‘…they hear “Africa” and they think that there can’t be any good services….and these are people in Georgetown [very affluent neighborhood in Washington DC] who travelled. It wasn’t like it was people who had never left, not like in the hinterland.’ (24^th^ Oct 14, Professor, Education Policy)

The significant generalization and over-simplification that in turn influences one’s perceptions of what is or is not possible lead people to hold, often firm, beliefs regarding the fundamental capacity of the country to deliver anything other than that which they expect from them:

‘I think that the starting position is we have nothing to learn from these people….the fact that in India, in selected circumstances, can deliver first world results in highly complicated case, at a fraction of the cost is simply dismissed as got to be sub-quality care…..when you are sure of something, you don’t have to explain it’ (15^th^ Oct 14, Professor, Public Health)

## Discussion

Our research highlights a particular problem in the Reverse Innovation process – that prior expectations, invoked by the LIC cue, are interfering with a transparent and objective learning process. The informants, experienced in transferring or attempting to transfer evidence from low-income countries into the US context, describe a prevalent application of stereotypes towards other contexts, but particularly low-income countries. Actors conflate local context with national context, and draw on flimsy country-level conceptualizations to predict whether what works in one context would or would not work in their own. This process may occur with any context perceived to be different to one’s own, but is more apparent with low-income countries because these are less familiar, and media representations are perhaps more stereotyped than other contexts. The criteria with which people judge one context to be or not be similar to one’s own seem varied and diverse, but do not stop people from making those judgments with some confidence. We expected informants to cite technical differences between LICs and HICs, for example disease profiles and case mix, but instead general national stereotypes were more frequently noted. Advancing on the ‘not-invented-here’ culture, we describe a ‘not-invented-in-a-context-perceived-to-be-similar-to-here’ culture. From an evidence-based medicine perspective, this is less than desirable. Generalizability requires a detailed knowledge of context so that the causal mechanisms that explain the relationship between intervention and outcome may be identified in one’s own context. Context includes the structures and processes, the actors and the institutions, that are involved in a change process and that partially explain the relationship between an intervention and an outcome. An intervention on its own is necessary but not sufficient for an outcome. The delivery model, how it was implemented, the specific barriers and challenges, are part of the process too. Considering that no two contexts are the same, it does not follow that an intervention successful (or unsuccessful) in one location will automatically be successful in another. It is necessary to ‘know deeply’ the innovator context so that any inferences regarding transferability of an innovation to one’s own context can be properly assessed. These local explanatory variables are complex and certainly cannot be inferred from national-level generalizations alone, however our informants’ experiences suggest that this is a common occurrence. We interviewed informants from education, social policy and international law, and found that this experience is not unique to healthcare. McGill et al. found that practitioners working in the design and management of the built environment at local government levels in the UK are more receptive to interventions that have been ‘delivered in a similar country’ – although do not discuss how these actors determine what does or does not constitute similar [30]. Our research suggests that ‘similarity’ is a flimsy, very personal construct based on conflated micro and macro contextual characteristics and that this leads to stereotyped generalizations that can interfere with the Reverse Innovation process.

Although individuals may have explicit biases, implicit biases are not tangible or tied to rational thought. Country level stereotypes contribute to rash generalizations, and these often-negative associations can appear outside of an individual’s conscious awareness [5]. In an attempt to translate the Brazilian Community Health Worker model in to the health system in Wales, Johnson et al. describe confronting issues of ‘pride’ and ‘prejudice’ during the persuasion stage with local stakeholders [31]. Harris et al. (forthcoming) have found that public health academics rate research differently as a result of the source, in some instances [32]. There has been an increased interest in the psychology field in measuring aspects of thinking that may not be overtly accessible to individual consciousness [33] and there is now an extensive literature on what is referred to as the “introspectively unidentified” [34]. Judging probability based on the essential features of a parent population is a heuristic, a mental short cut, which in all likelihood will be inaccurate and lead to false faith in some cases, and missed opportunities in other cases [35].

It is striking that although country of origin cues are considered highly important in the marketing industry, in healthcare research relatively little attention has been given to how individual actors perceive the quality and value of research or evidence from certain contexts. In healthcare research, much more emphasis is placed on the internal validity of the research, its conduct, risk of bias, and fidelity to research methodology, the structural and institutional ingredients to manage change and the characteristics of the innovation itself. The marketing industry has developed techniques of counter-stereotyping and branding to address potential biases towards certain sources [5]. Perhaps these techniques could be adopted in the health industry as well. Greenhalgh et al. note that context and confounders lie at the very heart of diffusion of complex innovations and are not extraneous to the object of study; they are an integral part of it [2]. Rogers touches on the idea of homophily as an impediment in the adopter context, a sociological concept that individuals have a tendency to associate with others of their same kind [36]. Homophily is heightened within healthcare organizations [37] and narrows an individual’s social world, leading to “herd” behavior [38, 39]. As a result of homophily, individuals within the healthcare sector who are most in need of innovation may actually be the least prone to adopt it [40]. Nonetheless, curiously absent is examination of the impact of actor perceptions of the innovator context. Considering that within countries, regions and cities have different economic, social and cultural landscapes, country-level generalizations hold even less merit.

This study is not without its limitations. Our informants are all from the US context only and we have not captured the views or experiences of front-line clinicians or service users as this was beyond the scope of the study and our resources. Although we have described some valuable insights that might stimulate academic debate we cannot generalize our findings to other contexts. Further research should be conducted in other high-income contexts, broadening also the pool of respondents to front-line clinicians and patients. Finally, our interviews were a mix of telephone and face-to-face interviews and it is possible that the different modes might alter the responses. There are, however, lessons to be learned by developed country health systems from experiences in developing countries, and opportunities for Reverse Innovation, where necessity and ingenuity have overcome resource constraints to achieve positive outcomes. We must allow ourselves to ‘know’ these contexts better before assuming they are too different for any learning to hold merit [41].

## Conclusion

Acknowledging the limitations of the research study, our findings suggest that those interested in Reverse Innovation should reflect carefully on the value of disclosing the source of the innovation that is being proposed, if doing so is likely to invoke negative stereotyping. There may be merit in adopting some techniques from the cognitive psychology and marketing literatures to understand better the relative importance of source in healthcare research and innovation diffusion and efforts to develop counter-stereotyping techniques may be useful in a diffusion of innovation process.
